# Determinants of Implanon Discontinuation among Women Who Use Implanon at Bahir Dar Town Health Institutions, Northwest Ethiopia, 2019: A Case-Control Study

**DOI:** 10.1155/2020/9048609

**Published:** 2020-09-24

**Authors:** Tsegaw Yehuala, Ergoye Melese, Kassawmar Angaw Bogale, Baye Dagnew

**Affiliations:** ^1^Department of Epidemiology and Biostatistics, School of Public Health, College of Medicine and Health Sciences, Bahir Dar University, P.O. Box 79, Bahir Dar, Ethiopia; ^2^Department of Human Physiology, School of Medicine, College of Medicine and Health Sciences, University of Gondar, P.O. Box 196, Gondar, Ethiopia

## Abstract

**Background:**

Implanon is a long-acting reversible contraceptive method that is 99% effective in preventing unintended pregnancy. Despite its effectiveness, the rate of Implanon discontinuation is high. In Ethiopia, there is limited information about determinants of Implanon discontinuation. Therefore, this study aimed to identify the determinants of Implanon discontinuation among women who used Implanon at Bahir Dar town health institutions.

**Methods:**

We employed an unmatched case-control study to find out the determinants of Implanon discontinuation at Bahir Dar town health institutions from March to June 2019 using the multistage stratified sampling technique to select study participants. Cases were women who had discontinued Implanon before completion of 3 years, and controls were women who had removed Implanon at the date of appointment (3 years). A pretested, structured questionnaire with face-to-face interviews was used. Binary logistic regression was performed to identify determinants of Implanon discontinuation. In the final model, variables with a *p* value of <0.05 were considered significant at 95% confidence interval and the strength of association was measured using odds ratio.

**Results:**

Primary education (AOR = 0.104, 95% CI (0.02–0.48)), secondary education (AOR = 0.48, 95% CI (0.24–0.952)), women who have no child (AOR = 2.04, 95% CI (1.2–3.4)), women who had no discussion with their partner (AOR = 2.2, 95% CI (1.39–3.57)), mass counseling (AOR = 3.5, 95% CI (1.75–7.01)), women who had no counseling about side effects (AOR = 1.7, 95% CI (1.07–2.07)), women who experienced side effects (AOR = 2.2, 95% CI (1.4–3.4)), and purpose of family planning use (AOR = 2.5, 95% CI (1.14–4.8)) were determinants of Implanon discontinuation.

**Conclusion:**

Implanon discontinuation is attributed by multifactorial involvement. Women's educational status, nulliparity, no counseling, not informed of side effects, and no partner discussion are significant factors. Health sector stakeholders need to tailor counseling services at individual level to bolster family planning utilization until the desired time.

## 1. Background

Progestin-only injectables and implants are highly effective, long-acting contraceptive methods used by most women [1]. Implanon is a hormone-releasing birth control method that is 99% effective in preventing unintended pregnancy and is used by women up to 3 years [2–4].

The main mechanism of action of Implanon is stopping the release of an egg from the ovary, thickening mucus in the cervix, which may prevent the sperm from reaching the egg, and changing the lining of the uterus [2, 5]. Although it involves minor surgical procedures, clients should get adequate counseling on general advantages, disadvantages, adverse effects, and their rights to discontinue Implanon use and a clarification on the rapid return to fertility once the Implanon is removed [6]. Newer-generation implants such as Implanon, Jadelle, Norplant, and Sino-implant are smaller and easily inserted and removed with smaller complications [7].

Over 4.5 million women have used Implanon worldwide [5]. In sub-Saharan Africa, approximately 14 million unintended pregnancies occur for poor use of contraceptives and there is a high discontinuation rate [8]. A high number of women become exposed to the risk of conception at three months after discontinuation, 40% or more women were at the risk of conception in Egypt, 42% in Ethiopia, 51% in Kenya, 73% in Malawi, 56% in United Republic of Tanzania, and 47% in Zimbabwe [9]. The Ethiopian Demographic and Health Survey (EDHS) 2016 reported that the implant users are only about 8% among all other method users and, in the five years preceding the survey, over one-third contraceptive users (35%) discontinued within 12 months. The Ethiopian Mini Demographic and Health Survey 2019 reported that the implant users are only about 9% [10]. Implanon discontinuation was 16% in Tigray, Ethiopia, which leads to higher rates of fertility rate, unwanted pregnancies, and induced abortion [11]. Experiencing side effects such as bleeding and also lack of/deficient preinsertion counseling are amongst the common risk factors for discontinuation of Implanon [12–15]. These might have social and economic consequences. However, there was paucity of study in Ethiopia about determinants of Implanon discontinuation. Therefore, this study aimed to identify the determinants of Implanon discontinuation among women who used Implanon in Bahir Dar town health institutions, Northwest Ethiopia, 2019.

## 2. Methods

### 2.1. Study Design, Area, and Period

An institution-based unmatched case-control study was employed at Bahir Dar town health institutions from March 1, 2019, to June 1, 2019. Bahir Dar town is the capital city of Amhara Region in Northwest Ethiopia, and it is 560 km away from Addis Ababa, the capital city of Ethiopia. According to the Bahir Dar town health department office report, the total population of Bahir Dar town is 364,339 and, in the town, there are 34 health institutions; among these, there are 2 government hospitals, 3 private hospitals, 10 government health centers, 1 private health center, and 10 medium private clinics, which provide family planning services including Implanon insertion and removal.

### 2.2. Source Population

#### 2.2.1. Cases

This includes all women of the reproductive age group (15–49) who requested Implanon removal in the Bahir Dar town health institutions before completion of three years in the study period.

#### 2.2.2. Controls

This includes all women of the reproductive age group (15–49) who requested Implanon removal in the Bahir Dar town health institutions at completion of three years in the study period.

### 2.3. Study Population

The study population included all women of the reproductive age group who requested Implanon removal in selected health institutions in the study period.

### 2.4. Eligibility Criteria

#### 2.4.1. Inclusion Criteria

All women in the selected health institutions who requested removal of Implanon before completion of three years during the study period were included as cases.

All women in the selected health institutions who requested removal of Implanon at the intended time (appointment date for removal) during the study period were included as controls. Women who requested removal of Implanon for conceiving/child birth in the study period were excluded.

### 2.5. Sample Size Determination and Sampling Procedure

No formal education, age <20 years, and had side effects were determinant factors of Implanon discontinuation in the previous study conducted in southern Ethiopia and Debre Markos [16, 17]. We used Epi Info version 7.2.2 to calculate sample size with the assumption of power 90%, control-to-case ratio of 3 : 1, 95% confidence level, design effect of 2, and finally 10% of nonresponse rate. The initial sample size was 254 (64 cases and 190 controls); then after adding 10% nonresponse rate, the final sample size was 141 cases and 418 controls with a total of 559 women. First, total health institutions in Bahir Dar town were identified and then categorized into governmental, private, and nongovernmental health institutions. Then, the multistage stratified sampling technique was applied. From thirty-four health institutions, thirteen were selected using the lottery method through which Implanon removal service was provided. From the average previous three months' data record of each selected health institutions, 1161 women (287 removals before recommended date and 874 removals at recommended date) have booked for Implanon removal and, among these, 122 women removed Implanon because of the reason that they wanted to conceive and exclude them for sample allocation purpose. Finally, the sample was proportionally drawn from each selected health institution based on the average delivery rate, three months prior to data collection.

In each selected health facility, the cases were selected consecutively until the required sample size was reached, and we selected three controls per case using the systematic sampling method. The control interval for each health facility was 2, and we used the lottery method to select the first client in each health facility..

### 2.6. Study Variables

#### 2.6.1. Dependent Variable

It includes Implanon discontinuation and coded as follows: 1 = Implanon discontinuation and 0 = recommended date.

#### 2.6.2. Independent Variables

Independent variables include sociodemographic variables (age in years, marital status, women's educational status, partner's educational status, religion and occupation, and husband's involvement), obstetric factors (number of children, abortion, and future plan of child birth), and method-related factors (weight gain, side effects, follow-up, counseling, discussion with their partners, and place of insertion).

### 2.7. Operational Definitions

#### 2.7.1. Implanon Discontinuation

It is discontinuation of the use of Implanon before completion of three years.

#### 2.7.2. Counseling

Counseling includes making the women aware of its long protection, effectiveness, and side effects due to insertion and removal.

#### 2.7.3. Side Effects

It is the development of at least one of the following conditions: menstrual disruption, headache, and insertion arm pain. 

### 2.8. Data Collection Procedure

We recruited 13 midwives who were working in the selected health institutions as data collectors after providing one-day training, and the principal investigator as supervisor was there to follow the data collection activity. The pretest was conducted in Addisalem Primary Hospital and Gamby General Hospital, which were out of the selected study facility before the actual data collection process.

### 2.9. Data Collection Tools

We used a structured, pretested questionnaire, adopted from EDHS 2016 and other published literature studies conducted in Wolaita Zone, Southern Ethiopia, and Bale Zone, Southeast Ethiopia [17, 18]. The questionnaire was first prepared in English and then translated to the local language (Amharic language) and backtranslated to English by language experts to ensure consistency. The questionnaire has three parts. The first part comprises the sociodemographic characteristics of mothers and their partners, the second part has obstetric and gynecological characteristics of women, and the third part has the role of partner and counseling service. Data collectors collected the data with face-to-face interviews using an Amharic version of the questionnaire.

### 2.10. Data Quality Control

We translated the questionnaire into Amharic language, which is the mother tongue in the study area, for easy communication and understanding. We gave one-day training for data collectors regarding the purpose of the study, data collection technique, and ethical concerns during data collection. The principal investigator strictly followed the overall activity of the data collection process. We conducted a pretest on 28 individuals. All returned questionnaires were examined for completeness and consistency throughout the data collection period.

### 2.11. Data Processing and Analysis

After coding, the data were entered into Epi Info version 7.2.2 and exported into Statistical Package for Social Science (SPSS) version 23 for analysis. Frequency was used to describe the characteristics of the study. Bivariable logistic regression analysis was performed to select candidate variables for multivariable binary logistic regression analysis. Those variables with a *p* value of ≤0.2 were entered into the final model. The multivariable logistic regression model was fitted using the backward elimination technique. Odds ratio with 95% confidence interval was used to measure the strength of association, and those variables having *p* value <0.05 were considered significant.

## 3. Results

### 3.1. Sociodemographic Characteristics of Study Participants

A total of 559 (141 cases and 418 controls) participants took part in the study with a response rate of 100%. The mean age of the case and control mothers was 26.87 (SD = ±4.98) and 28.51 (SD = ±4.8) years, respectively. One hundred and sixty-one (38.5%) control mothers and 44 (31.2%) cases were found to be in the age category of 25–29 years. Among the total, 102 (72.3%) cases and 286 (68.4%) controls attained a certificate and above. From the partner's educational status, 69 (48.9%) cases and 206 (68.4%) controls attained a certificate and above. Regarding the occupational status of women, forty-nine (8.8%) cases and 193 (34.5%) controls were employed (Table 1).

### 3.2. Obstetric- and Gynecological-Related Characteristics of Study Participants

This study showed that 357 (85.4%) controls and 95 (67.4%) cases had children during insertion of Implanon. The future plan of the study participants among cases 133 (94.3%) and controls 363 (86.8%) was needed to space child birth, and others were needed to limit child birth. Among the total study participants, 116 (82.3%) cases and 373 (89.2%) controls have no history of abortion (Table 2).

### 3.3. Role of the Partner and Counseling Services' Characteristics of Controls and Cases in Bahir Dar Town Health Institutions

About 70 (49.6%) cases and 261 (62.4%) controls had an individual counseling service before inserting Implanon, and 30 (21.3%) cases and 22 (5.3%) controls did not get any type of counseling before inserting Implanon. Among those who have used Implanon, 75 (53.2%) cases and 320 (76.6%) controls had a discussion with their partners. Those who used Implanon by themselves were 85 (60.3%) cases and 259 (61.96%) controls. Among the study participants, 84 (59.6%) cases and 217 (51.9%) controls got Implanon insertion at the health center.

Forty-six (32.6%) cases and 115 (27.5%) controls had follow-up appointment during the Implanon utilization period. Among the cases, 84 (59.6%) experienced side effects, and 139 (33.3%) controls also developed a side effect. Among women who developed side effects, menstrual disruption was the most common side effect for both cases (39.7%) and controls (12.2%), and other side effects were headache 32 (22.7%) and 36 (8.6%), irritability/restlessness 30 (21.3%) and 25 (6%), weight gain 17 (12.1%) and 36 (8.6%), and amenorrhea 16 (8.6%) and 47 (11.2%) for cases and controls, respectively (Table 3).

### 3.4. Determinants of Implanon Discontinuation

After adjusting other variables in a multivariable logistic regression analysis, women's educational status, absence of children, no counseling, purpose/future plan of Implanon use, did not get counseling about side effects, experiencing side effects, and no discussion with their partners were determinants of Implanon discontinuation.

Women who had attended primary and secondary education level were 89.6% less likely to discontinue Implanon as compared to those who had a certificate and above educational level (AOR = 0.104, 95% CI (0.02–0.48)) and (AOR = 0.48, 95% CI (0.24–0.952)), respectively. The study also revealed that the odds of Implanon discontinuation was 2.04-fold higher among women who had not had children compared to their counterparts (AOR = 2.04, 95% CI (1.2–3.4)). There was 2.24-fold higher odds of Implanon discontinuation among women who had not discussed with their partners than those discussed with their partner (AOR = 2.2, 95% CI (1.39–3.57)). The odds of Implanon discontinuation was 3.5 times higher among women who had got mass counseling during the insertion of Implanon than those who had got individual counseling service (AOR = 3.5, 95% CI (1.75–7.01)). Women who did not get counseling on side effects of Implanon insertion were 1.7 times more likely to discontinue than those who got counseling about side effects during insertion of Implanon (AOR = 1.7, 95% CI (1.07–2.7)). The odds of discontinuing Implanon were 2.2-folds higher among women who experienced side effects as compared to those who were not experiencing side effects (AOR = 2.2, 95% CI (1.4–3.4)). The odds of discontinuing Implanon were 2.5 times higher among women who want to space children than those women who want to limit child birth (Table 4).

## 4. Discussion

The odds of Implanon discontinuation in this study were reduced by 90% and 52% among women who had attended primary and secondary education as compared with those who had a certificate and above, respectively. This finding is in line with studies conducted in Debre Markos town, Ethiopia [16]. These might be because women who had higher education level might seek healthcare providers sooner than less-educated women. Other studies conducted in Wolaita, Southern Ethiopia, and Bangladesh reported a higher Implanon discontinuation among women who had no formal education as compared to women who had attended secondary and above educational level [17, 19]. However, there are studies in Debre Tabor [20] and Bale zone of Ethiopia [18], which concluded that education was not associated with Implanon discontinuation.

The odds of Implanon discontinuation among women who had not had children during insertion were 2.04 times than those who had children during insertion. This finding is consistent with other studies, as observed in Bangladesh, Burkina Faso, and Debre Tabor [7, 19, 20]. This might be because women who had not had children may have great intention to discontinue Implanon to get a child. Mass counseling was also a determinant factor for Implanon discontinuation. The odds of Implanon discontinuation among women who had got mass counseling in this study were 3.5 times higher than those who got counseling individually (AOR = 3.5, 95% CI (1.2–3.4)). The reason might be that mass counseling could lead to inadequate delivery of information and incomplete understanding. However, a study conducted in Wolaita zone, Ethiopia, showed no association between types of counseling with Implanon discontinuation [17]. The reason might be variations in commitment, the way how to counsel, and the different skills of one healthcare provider to another. Participants who did not get preinsertion counseling had higher Implanon discontinuation than those who get counseling, which is supported by another study in Egypt [21]. This might be due to the fact that women who did not get counseling about the Implanon position, side effects, or other related issues are more likely to remove it when started to develop side effects or due to social influences.

Not discussing with a partner was also a determinant for Implanon discontinuation. The odds of Implanon discontinuation among women who had not discussed with their partner during insertion of Implanon were 2.2 times more likely than those who had discussed with their partner. This might be due to differences in a conflict of interest of partners to have additional children that leads to discontinuation. This is in line with studies conducted in Bale zone, Ethiopia [18].

Presence of side effects was associated with Implanon discontinuation. The odds of Implanon discontinuation among women who had experienced a side effect were 2.2 times more likely when compared to those women who had not experienced a side effect. This finding was in agreement with previously published works [11, 16, 20–25]. The reason might be fear, discomfort, and other physiological and psychological problems induced by side effects. Another reason might also be because of inadequate preinsertion counseling by healthcare providers about possible side effects. The odds of Implanon discontinuation among women who had not counseled about side effects were 1.7 times more likely than those who had counseled about side effects. This finding was supported by other studies [11, 20, 21, 23]. This might be because of inadequate counseling about the side effects by healthcare providers that delivered the services. The odds of discontinuing Implanon were 2.5 times more likely among women who wanted to space children than those women who wanted to limit child birth. These findings were supported by studies conducted in Bale zone [18]. These might be for improper choice of family planning.

## 5. Limitations of the Study

Because of the nature of the design, there might be recall bias and information bias. The side effects were not labeled in severity. Another limitation is that the study was institution based, so it might not be generalized.

## 6. Conclusions

The determinants of Implanon discontinuation in the current study were women's educational status, absence of children, no counseling, not informed about the side effects, absence of discussion with their partners, and had side effects. The aforementioned findings demand the involvement of health sector stakeholders to provide preinsertion counseling according to the national guideline for family planning services by giving emphasis on possible expected side effects and consider about counseling on their partners.

## Figures and Tables

**Table 1:** Sociodemographic characteristics of study participants among women who use Implanon in Bahir Dar town health institutions, Northwest Ethiopia, December 2019.

Variables	Categories	Cases (*n* = 141), *n* (%)	Controls (*n* = 448), *n* (%)
Age of respondents in years	<20	12 (8.5%)	9 (2.2%)
	20–24	35 (24.8%)	89 (21.3%)
	25–29	44 (31.2%)	161 (38.5%)
	30–34	38 (27%)	109 (26.1%)
	≥35	12 (8.5%)	50 (12%)

Marital status	Single	50 (35.5%)	138 (33%)
	Married	75 (53.2%)	253 (60.5%)
	Widowed	8 (5.7%)	10 (2.4%)
	Divorced	8 (5.7%)	16 (3.8)

Religion	Orthodox	97 (68.8%)	260 (62.2%)
	Muslim	21 (14.9%)	96 (23%)
	Protestant	23 (16.3%)	61 (14.6%)

Women's education status	No education	20 (14.2%)	40 (9.6%)
	Primary education	2 (1.4%)	30 (7.2%)
	Secondary education	17 (12.1%)	62 (14.8%)
	Certificate and above	102 (72.3%)	286 (68.4%)

Partner's educational status	No education	34 (24.2%)	88 (21.1%)
	Primary education	7 (5%)	19 (4.5%)
	Secondary education	31 (22%)	105 (25.1%)
	Certificate and above	69 (48.9%	206 (68.4%)

Maternal's occupational status	Housewife	23 (16.3%)	71 (17%)
	Farmer	9 (6.4%)	8 (1.9%)
	Merchant	35 (24.8%)	99 (23.7%)
	Employed	49 (34.8%)	193 (46.2%)
	Others (students)	25 (17.7%)	46 (11%)

**Table 2:** Obstetric and gynecological characteristics of study participants among women who use Implanon in Bahir Dar town health institutions, Northwest Ethiopia, December 2019.

Variables		Cases (*n* = 141), *n* (%)	Controls (*n* = 448), *n* (%)
Having children	No	46 (32.6%)	61 (14.6%)
	Yes	95 (67.4%)	357 (85.4%)

No. of children	Less than four children	104 (95.4%)	331 (92.7%)
	Four and above	5 (4.6%)	26 (7.3%)

Future plan	Space child birth	133 (94.3%)	363 (86.8%)
	Stop child birth	8 (5.7%)	55 (13.2%)

History of abortion	No	116 (82.3%)	373 (89.2%)
	Yes	25 (17.7%)	45 (10.8%)

Partner want to be pregnant in the next two years	No	81 (57.4%)	230 (55%)
	Yes	60 (42.6%)	188 (45%)

**Table 3:** Characteristics of study participants regarding counseling and other variables, Bahir Dar, Northwest Ethiopia, December 2019.

Variables		Categories	Cases (*n* = 141), *n* (%)	Controls (*n* = 448), *n* (%)
Types of counseling provided		Individual counseling	70 (49.6%)	261 (62.4%)
		Mass counseling	20 (14.2%)	26 (6.2%)
		With partner	21 (14.9%)	109 (26.1%)
		No counseling	30 (21.3%)	22 (5.3%)

Counseling about	Advantage	Yes	85 (60.3%)	276 (66%)
		No	56 (39.7%)	142 (34%)
	Side effect	Yes	41 (29.1%)	205 (49%)
		No	100 (70.9%)	213 (51%)
	Effectiveness	Yes	48 (34%)	197 (47.1%)
		No	93 (66%)	221 (52.9%)
	When to insert and remove	Yes	73 (51.8%)	215 (51.4%)
		No	68 (48.2%	203 (48.6%)

Discussion with partner		Yes	75 (53.2%)	320 (76.6%)
		No	66 (46.8%)	98 (23.4%)

Who accompanied with you?		Husband	38 (27%)	137 (32.8%)
		Mother	4 (2.8%	14 (3.3%)
		Nobody	93 (66%)	258 (61.7%)
		Others	6 (4.3%)	9 (2.2%)

Who was a decision maker to use Implanon?		Wife	85 (60.3%)	259 (61.96%)
		Husband/partner	25 (17.7%)	64 (15.31%)
		Healthcare provider	31 (22%)	95 (22.73%)

Place of insertion		Health center	84 (59.6%)	217 (51.9%)
		Health post	7 (5%)	23 (5.5%)
		Hospital	30 (21.3%)	93 (22.2%)
		NGO and private clinic	20 (14.2%)	85 (20.3%)

Why did you use Implanon?	Low failure rate	No	82 (58.2%)	198 (47.4%)
		Yes	59 (41.8%)	220 (52.6%)
	Low follow-up	No	61 (41.3%)	144 (34.4%)
		Yes	80 (56.7%)	274 (65.6%)
	Less side effects	No	119 (84.4%)	279 (66.7%)
		Yes	22 (15.6%)	139 (33.3%)
	Lack of other methods	No	122 (86.5%	387 (92.6%)
		Yes	19 (13.5%)	31 (7.4%)
	Useful for a long period	No	64 (45.4%)	191 (45.7%)
		Yes	77 (54.6%)	227 (54.3%)

Experiencing a side effect		Yes	84 (59.6%)	139 (33.3%)
		No	57 (40.4%)	279 (66.7%)

What type of side effect?	Menstrual bleeding	Yes	56 (39.7%)	51 (12.2%)
		No	85 (60.3%)	367 (87.8%)
	Weight gain	Yes	17 (12.1%)	36 (8.6%)
		No	124 (87.9%)	382 (91.4%)
	Headache	Yes	32 (22.7%)	36 (8.6%)
		No	109 (77.3%)	382 (91.4%)
	Irritability/restlessness	Yes	30 (21.3%)	25 (6%)
		No	111 (78.7%)	393 (94%)
	Menstrual cycle stops	Yes	16 (11.3%)	47 (11.2%
		No	125 (88.7%)	371 (88.8%)

Follow-up after insertion		Yes	46 (32.6%)	115 (27.5%)
		No	95 (67.4%)	303 (72.5%)

Satisfied by healthcare provider		No	70 (49.6%)	170 (40.7%)
		Yes	72 (50.4%)	248 (59.3%)

**Table 4:** Determinant of Implanon discontinuation of study participants among women who use Implanon in Bahir Dar town health institutions, Northwest Ethiopia, December 2019.

Variables		Implanon discontinuation		COR (95% CI)	AOR (95% CI)
		Cases (*N*, %)	Controls (*N*, %)		
Women's educational level					
	No education	20 (14.2%)	40 (9.6%)	1.402 (0.78, 2.5)	0.76 (0.39, 1.5)
	Primary education	2 (1.4%)	30 (7.2%)	0.187 (0.04, 0.79)	0.1 (0.02, 0.48)^*∗*^
	Secondary education	17 (12.1%)	62 (14.8%)	0.77 (0.43, 1.37)	0.48 (0.24, 0.95)^*∗*^
	Certificate and above	102 (72.3%)	286 (68.4%)	1	1

Purpose of using Implanon					
	Space child birth	133 (94.3%)	363 (86.8%)	2.5 (1.17, 5.4)	2.5 (1.14, 5.78)^*∗*^
	Stop child birth	8 (5.7%)	55 (13.2%)	1	1

Having children	No	46 (32.6%)	61 (14.6%)	2.8 (1.8, 4.4)	2.04 (1.2, 3.4)^*∗*^
	Yes	95 (67.4%)	357 (85.4%)	1	1

Types of counseling					
	Mass counseling	20 (14.2%)	26 (6.2%)	2.86 (1.51, 5.43)	3.5 (1.75, 7.01)^*∗∗∗*^
	With partner	21 (14.9%)	109 (26.1%)	.71 (0.42, 1.22)	0.84 (0.47 ,1.5)
	No counseling	30 (21.3%)	22 (5.3%)	5.08 (2.76, 9.35)	2.34 (1.14, 4.8)^*∗*^
	Individual counseling	70 (49.6%)	261 (62.4%)	1	1

Discussion with partner	No	66 (46.8%)	98 (23.4%)	2.87 (1.92, 4.29)	2.2 (1.39, 3.57)^*∗∗∗*^
	Yes	75 (53.2%)	320 (76.6%)	1	1

Counseling side effects	No	100 (70.9%)	213 (51%)	2.34 (1.55, 3.54)	1.7 (1.07, 2.7)^*∗*^
	Yes	41 (29.1%)	205 (49%)	1	1

Experienced side effects	Yes	84 (59.6%)	139 (33.3%)	2.95 (1.99, 4.38)	2.2 (1.4, 3.4)^*∗∗∗*^
	No	57 (40.4%)	279 (66.7%)	1	1

## Data Availability

The dataset is accessible from the corresponding author upon rational request.

## Abbreviations

AOR: Adjusted odds ratio
CI: Confidence interval
COR: Crude odds ratio
EDHS: Ethiopian Demographic and Health Survey
EPI Info: Epidemiological information.
